# Graduate training, credentialing, and continuing education to prepare genetic counselors for laboratory roles—Results of a national survey

**DOI:** 10.1002/jgc4.1883

**Published:** 2024-02-09

**Authors:** Lisa Schwartz, Mia S. Mackall, Aishwarya Arjunan, McKinsey Goodenberger, Rachel Mills, Chloe Ham, Sarah Witherington

**Affiliations:** ^1^ Department of Biomedical Laboratory Sciences The George Washington University Ashburn Virginia USA; ^2^ Clinical Genetic Services Natera, Inc. Austin Texas USA; ^3^ Department of Medical Affairs GRAIL, LLC. Menlo Park California USA; ^4^ Division of Laboratory Genetics and Genomics Mayo Clinic Rochester Minnesota USA; ^5^ MS Genetic Counseling Program University of North Carolina Greensboro Greensboro North Carolina USA; ^6^ MD Program, Sidney Kimmel Medical College Thomas Jefferson University Philadelphia Pennsylvania USA; ^7^ Oncology Genetic Services BioReference Health, LLC Elmwood Park New Jersey USA

**Keywords:** education, genetic counselors, professional development

## Abstract

Opportunities for genetic counselors to work in a variety of practice settings have greatly expanded, particularly in the laboratory. This study aimed to assess attitudes of genetic counselors working both within and outside of the laboratory setting regarding (1) the re‐wording and/or expansion of key measures of genetic counselors' competency, including practice‐based competencies (PBCs) and board examination, to include laboratory roles, (2) preparation and transferability of competencies developed in master's in genetic counseling (MGC) programs to different roles, (3) need of additional training for genetic counselors to practice in laboratory settings, and (4) preferred methods to obtain that training. An e‐blast was sent to ABGC diplomats (*N* = 5458) with a link to a 29‐item survey with 12 demographic questions to compare respondents to 2021 NSGC Professional Status Survey (PSS) respondents. Statistical comparisons were made between respondents working in the laboratory versus other settings. Among 399 responses received, there was an oversampling of respondents working in the laboratory (52% vs. 20% in PSS) and in non‐direct patient care positions (47% vs. 25% in PSS). Most respondents agreed the PBCs were transferable to their work yet favored making the PBCs less direct patient care‐focused, expanding PBCs to align with laboratory roles, adding laboratory‐focused questions to the ABGC exam, and adding laboratory‐focused training in MGC programs. Most agreed requiring post‐MGC training would limit genetic counselors' ability to change jobs. Genetic counselors working in the laboratory reported being significantly less prepared by their MGC program for some roles (*p <* 0.001) or how the PBCs applied to non‐direct patient care positions (*p <* 0.001). Only 53% of all respondents agreed that NSGC supports their professional needs and others in their practice area, and genetic counselors working in the laboratory were significantly less likely to agree (*p* = 0.002). These sentiments should be further explored.

**What is known about this topic**

- Nearly one‐third of genetic counselors (GCs) are working in the laboratory setting.
- The 2019 ACGC Practice‐Based Competencies (PBCs) do not fully address the expanding roles of genetic counselors working in the laboratory, as found through a qualitative study of genetic counselors working in the laboratory, reported elsewhere.

**What this paper adds to the topic**

- Surveyed genetic counselors felt the 2019 ACGC Practice‐Based Competencies (PBCs), while translatable to genetic counselors' roles in the laboratory, should be rephrased and expanded to account for the varied, often non‐direct patient care, roles of genetic counselors.
- Genetic counselors working in the laboratory report having been less supported by their master's in genetic counseling program when they were considering working in a non‐direct patient care setting and feel less prepared for roles deemed important in the laboratory, including variant interpretation, limitations of genomic testing, and the business of health care, than genetic counselors working in other settings. They were also significantly less likely to agree that NSGC supports their needs and other genetic counselors in their area of practice.

## 1 INTRODUCTION

Numerous authors have reported on the emergence of new roles for genetic counselors in the laboratory setting (Cho & Guy, 2019; Christian et al., 2012; Goodenberger et al., 2017; Kotzer et al., 2014; McWalter et al., 2018; Swanson et al., 2014; Waltman et al., 2016; Zetzsche et al., 2014). However, studies regarding the perceived need for additional training to work in the laboratory setting have primarily focused on genetic counselors already working in these roles (Christian et al., 2012; Strohmeyer et al., 2023; Zetzsche et al., 2014) and not the broader genetic counselor community.

Key professional organizations to the field of genetic counseling, namely the Accreditation Council for Genetic Counseling (ACGC), the American Board of Genetic Counseling (ABGC), and the National Society of Genetic Counselors (NSGC), work in concert to establish standards, evaluate competency, and support the training of genetic counselors. As recently as 2020, the ACGC appointed a task force to review the then‐current version of the ACGC's Practice‐based Competencies (PBCs; ACGC, 2019a) using a survey of relevant stakeholders. Once new PBCs and Standards are released by ACGC, master's in genetic counseling (MGC) programs are expected to review and revise their curricula within 1 year to ensure they remain in compliance with the Standards and 2 years with the PBCs, and thus serve as key partners ensuring the training and competency of entry‐level genetic counselors.

The need for training of genetic counselors to evolve with advances in genomic testing and the changing workforce has been the topic of commentaries within the genetic counseling literature (Amendola et al., 2021; Hooker et al., 2014; Riconda et al., 2018; Swanson et al., 2014; Wickland & Trepanier, 2014). Amendola et al. (2021) offered recommendations for how the availability of genetic counseling can be expanded in the growing era of genomic medicine, including in areas of primary care and consumer‐driven genetic testing. They noted that a major challenge to increasing the number of genetic counselors trained are the relatively small size and number of master's‐level genetic counseling programs, which are limited by fieldwork placements where only cases involving direct patient care may be included in documentation for ABGC certification eligibility. Amendola et al. (2021) recommended that ACGC consider how cases involving non‐direct patient care, like those that may be obtained in the laboratory setting under the supervision of a genetic counselor, can be among the core cases counted towards eligibility for ABGC certification. Swanson et al. (2014) and Berg et al. (2018) have made similar recommendations. Yet outside of the work conducted by the ACGC and ABGC as noted above, to our knowledge, practicing genetic counselors' perceptions regarding revision or expansion of the PBCs and ABGC certification examination, and preferred methods by which genetic counselors may develop competencies needed for roles outside of the direct patient care setting, had not been previously explored. This study was intended to fill this gap in research.

The presented research is the second phase of an exploratory, mixed methods (QUAL→quant) study. During the first phase, Schwartz et al. (2023) conducted a qualitative study of 20 genetic counselors working in the laboratory and five MD or PhD non‐genetic counselor laboratory directors aimed at identifying competencies required, and perceived level of preparation of these competencies, for entry‐level genetic counselors working in the laboratory. The perceived value of additional credentialing for genetic counselors practicing in the laboratory setting was also explored. Genetic counselors working in the laboratory felt that the competencies obtained during their master's‐level training prepared them well to transition into this setting without the need of additional formalized training, and that instead additional skills needed to work in the laboratory setting were often developed through on‐the‐job training and interdisciplinary collaboration (Schwartz et al., 2023). Aligned with Swanson et al. (2014) and Amendola et al. (2021), the majority of participants recommended more exposure to diverse roles in genetic counseling programs' didactic and field training. An unexpected finding by Schwartz et al. (2023) was that several genetic counselors working in the laboratory questioned their professional identity as genetic counselors given that they were no longer providing direct patient care.

The second phase of the mixed methods study built upon the findings of Schwartz et al. (2023). Through development and administration of a quantitative survey, attitudes of genetic counselors working both within and outside of the laboratory setting regarding (1) the re‐wording and/or expansion of key measures of genetic counselors' competency, including practice‐based competencies (PBCs) and board examination, to include laboratory roles, (2) preparation and transferability of competencies developed in MGC programs to different roles, (3) need of additional training for genetic counselors to practice in laboratory settings, and (4) preferred methods to obtain that training were assessed. Questions regarding professional identity and perception of the NSGC as a community of practice were also included.

## 2 METHODS

### 2.1 Participants and procedures

Participants were recruited through an email with a link to an electronic survey, which was distributed on November 11, 2021 to 5458 diplomats of the ABGC. Recruitment announcements were also sent via the LinkedIn and Twitter accounts of the first author (LS) using genetic counselor‐related hashtags. The survey remained open until December 1, 2021. The survey was completed by 399 participants; incomplete surveys were not included in the analyses.

This study was reviewed and granted an exemption by The George Washington University Institutional Review Board (NCR203188). Implied informed consent was obtained for individuals who voluntarily completed the online survey and submitted their responses.

### 2.2 Instrumentation

Development of the 29‐item survey was informed by the findings of a qualitative study of experts in the diagnostic laboratory, including laboratory genetic counselors and non‐genetic counseling‐trained (MD/PhD) directors (Schwartz et al., 2023). The survey was finalized in consultation among the advisory board of the qualitative study and an educational research expert and was pilot‐tested by three non‐genetic counselors who were not involved with the study. The survey was built and distributed using Qualtrics©. Definitions of “laboratory genetic counselor” and “ACGC Practice‐Based Competencies (PBCs)”, along with a link to the 2019 PBCs on the ACGC website, were provided at the beginning of the survey.

The survey was divided into six sections, and each of the 29 items in Sections 1–5 included a 5‐point Likert scale (Strongly Disagree, Disagree, Neutral, Agree, Strongly Agree). Section 1 included questions regarding attitude towards the ACGC PBCs and ABGC board examination being changed to better reflect roles of genetic counselors working in the laboratory setting. Section 2 included 11 questions focused on perceptions of preparation for genetic counselor roles in the laboratory within MGC programs. Section 3 focused on additional training or credentialing for laboratory genetic counseling specialization. Section 4 focused on one's perception of identity as a genetic counselor, and Section 5 focused on the concept of communities of practice as a method of training for laboratory genetic counseling specialization.

Section 6 included an additional 12 demographic questions, several of which were similar to those in the 2021 NSGC Professional Status Survey (PSS; NSGC, 2021). Demographic information collected included academic degrees completed or in progress; participation in activities for competency development since completing a MGC program; range of years in which graduated from a genetic counseling program; status of ABGC certification and licensure; current position (direct patient care, non‐direct patient care, or mixed), primary employment setting, job title, roles, and primary area of practice; total number of years employed in current position and current area of practice; and whether or not one's current position required graduation from a MGC program, board certification, licensure, and clinical experience. No identifiable information was collected to ensure anonymity of the respondents. See Appendix S1 for the complete survey.

### 2.3 Data analysis

Descriptive statistics were used to compare the demographics of the survey respondents with those of the 2021 NSGC PSS. Responses to the demographic question regarding current primary employer work setting were used to create a new categorical variable, Lab_NonLab, with respondents indicating their employer as one of three diagnostic laboratory settings (non‐commercial/academic, commercial/academic, commercial/non‐academic) designated as Lab and the remainder of response options designated as NonLab. Chi Square and MANOVA using IBM SPSS Statistics 26 were conducted to compare Lab versus Non‐Lab respondents' responses on select demographic variables and the 29‐item Likert scale s, respectively.

## 3 RESULTS

### 3.1 Demographics

Of the 399 respondents, 388 (97.24%) had a master's degree in genetic counseling but several had completed or were in the process of completing other degrees (see Table 1). The vast majority (*n* = 274; 69.19%) of respondents were both ABGC‐certified and licensed, whereas 29.29% (*n* = 116) were ABGC‐certified but not licensed. Six respondents (1.52%) indicated that they were ABGC eligible, and three respondents did not respond to the question asking about certification and licensure status.

**TABLE 1:** Academic degrees completed or in the process of completing among respondents (*N* = 399).

Academic degree	Number indicating yes (% of total respondents)^a^
Master's degree (or equivalent) in Genetic Counseling	388 (97.24%)
Master's degree (or equivalent) in another life science‐based field (e.g., medical laboratory sciences, microbiology)	63 (15.79%)
Master's degree in Public Health (MPH)	34 (8.52%)
Master's degree in Education	36 (9.02%)
Master's degree in Business (MBA) or equivalent	37 (9.27%)
Medical degree (MD or DO)	35 (8.77%)
Doctoral degree in life science‐based field (e.g., biochemistry, genetics)	41 (10.28%)
Doctoral degree in education	32 (8.02%)
Other	38 (9.52%)

^a^ Participants could select more than one option, percentages will sum to >100.

Compared to the 2021 NSGC PSS, there was an oversampling of respondents working in the laboratory (52% vs. 20% in the PSS) and in non‐direct patient care positions (47% vs. 25% in PSS). Among genetic counselors working in the laboratory (*n* = 207), 71% (*n* = 147) were in non‐direct patient care positions. Among all respondents in non‐direct patient care positions (*n* = 186), 79% (*n* = 147) worked in a laboratory. Other sample demographics were similar to the 2021 PSS (see Table 2).

**TABLE 2:** Demographic information of survey respondents (*N* = 399).

Survey question—Section 6—Demographics	Survey respondents	
	*n*	%
Graduation year from MGC program		
2020–2021	42	10.53
2010–2019	197	49.37
2000–2009	112	28.07
1990–1999	29	7.27
1980–1989	15	3.76
1970–1979	2	0.50
n/a—Did not attend a MGC program	1	0.25
Current position type		
Direct patient care	133	33.33
Non‐direct patient care	186	46.62
Mixed position	79	19.80
Current primary employer work setting		
Diagnostic Laboratory—Non‐commercial, academic	30	7.52
Diagnostic Laboratory—Commercial, academic	37	9.27
Diagnostic Laboratory—Commercial, non‐academic	140	35.09
Hospital/Medical Facility—Academic Medical Center	81	20.30
Hospital/Medical Facility—Private (nonprofit or for profit)	38	9.52
Hospital/Medical Facility—Public	12	3.01
Pharmaceutical Company	13	3.26
Private Company	9	2.26
University	9	2.26
All others	42	<2.00
Current job title		
Genetic counselor	185	46.37
Genetic counselor, Senior/Lead/Supervisor/Coordinator	41	10.28
Medical Science Liaison	22	5.51
Laboratory Genetic Counselor/Coordinator/Support	23	5.76
Manager, Clinical Services/Genetic Services/Genetic Counseling	24	6.02
All others	102	<5.00
Roles within your current position^a^		
Patient‐facing/Direct Patient Care	178	44.61
Education/Teaching	231	57.89
Supervision—Students	211	52.88
Coordination—Clinical	105	26.32
Research	151	37.84
Supervision—Employees	84	21.05
Program Development	85	21.30
Writing—Education/Patient Material	165	41.35
Writing—Scientific	149	37.34
Laboratory—Support/Customer Service	146	36.60
Clerical Laboratory—Report Writing	89	22.30
Customer Liaison	96	24.01
Laboratory—Variant Interpretation	99	24.81
Management—Project	89	22.30
All others	718	<20.00
Current primary area of practice		
Cancer Genetics—Adult	79	19.80
Prenatal	48	12.03
Pediatrics	31	7.52
Molecular/Cytogenetics/Biochemical Testing	76	19.05
All others	162	<15.00
Total number of years employed in your current position		
Less than 1 year	74	18.55
1–4 years	151	37.84
5–9 years	120	30.01
10–14 years	27	6.77
15–19 years	15	3.76
20–24 years	6	1.50
More than 25 years	4	1.00
Total number of years in your current area of practice		
Less than 1 year	39	9.77
1–4 years	118	29.57
5–9 years	131	32.83
10–14 years	57	14.29
15–19 years	34	8.55
20–24 years	8	2.01
More than 25 years	11	2.76

^a^ Participants could select more than one option, percentages will sum to >100.

Respondents were asked which activities they had participated in to further develop the competencies needed to perform their job since completing their MGC program. The vast majority (*n* = 371; 93.00%) of respondents indicated that they had participated in continuing education rather than academic, credit‐bearing degrees (*n* = 60; 15.03%) or coursework (*n* = 76; 19.29%). As reported by participants in the qualitative study by Schwartz et al. (2023), a high percentage of respondents noted developing further competencies on the job, either as structured training (*n* = 351; 87.97%), informally by learning from others in the workplace (*n* = 364; 91.23%), or on their own (*n* = 378; 94.74%).

The survey included four questions aimed to assess requirements for respondents' genetic counseling positions. The responses, as compared between genetic counselors in the laboratory (Lab) and those in other work settings (NonLab), are presented in Table 3.

**TABLE 3:** Comparison of genetic counselors working in the laboratory and those in other work settings in regards to certification and/licensure status and current job requirements.

Survey question #12	Lab (%)	NonLab (%)	Chi‐Square
My current position required that I graduated from an accredited Master's in Genetic Counseling Program.	82.12%	93.12%	*X* ^2^ (3, *N* = 398) = 20.53, *p* = 0.00*
My current position required that I be board certified as a genetic counselor.	76.33%	86.39%	*X* ^2^ (3, *N* = 398) = 11.25, *p* = 0.01*
My current position required that I be licensed as a genetic counselor.	48.79%	52.36%	*X* ^2^ (3, *N* = 398) = 1.91, *p* = 0.59
My current position required that I had clinical experience after completing my Master's in Genetic Counseling Program	39.61%	27.74%	*X* ^2^ (3, *N* = 398) = 9.97, *p* = 0.02*

* Significant at *p* < 0.05.

Genetic counselors working in the laboratory were statistically less likely to report that their current position required they had graduated from an accredited master's in genetic counseling program [*X* ^2^ (3, *N* = 398) = 20.53, *p* < 0.001] or they that be board certified as a genetic counselor [*X* ^2^ (3, *N* = 398) = 11.25, *p* = 0.01]. However, it was more likely that their position required previous clinical experience than those respondents who worked in other work settings [*X* ^2^ (3, *N* = 398) = 9.97, *p* = 0.02]. There was no significant difference in licensure being a job requirement between genetic counselors working in the laboratory or other work settings [*X* ^2^ (3, *N* = 398) = 1.91, *p* = 0.59].

### 3.2 Preparation and practice of genetic counselors

**FIGURE 1:**
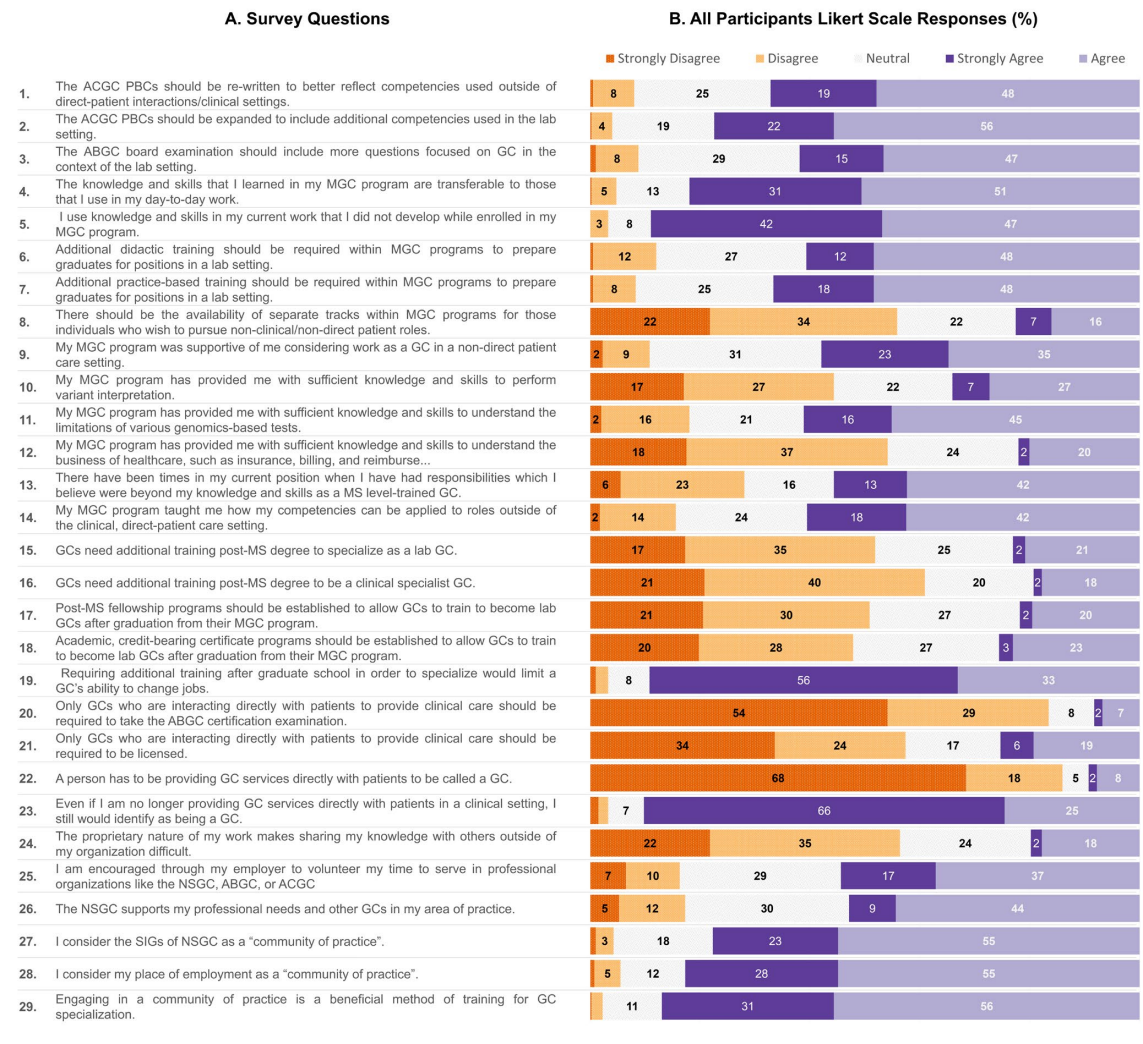
Overall Likert scale scores for all participants by survey question.

Descriptive and inferential statistics were used to describe the results of the Likert scale questions in the survey and compare the responses between genetic counselors working in the laboratory setting to those genetic counselors working in other practice settings. Figure 1 shows the results of the overall Likert scale scores, based on percentage who strongly disagree to strongly agree, for all participants, and Table 4 shows the comparisons of mean Likert scale scores between genetic counselors working in the laboratory setting (Lab) and those working in other settings (NonLab). Additional details are provided in the text below.

**TABLE 4:** Comparison of mean Likert scores between Lab and NonLab respondents.

Survey question	Mean Likert score
	Lab mean (SD)
	NonLab mean (SD)
	Mean difference (Lab mean − NonLab mean)
	*p*‐value
Section I—PBCs and ABGC exam	
1. The ACGC PBCs should be re‐written to better reflect competencies used outside of direct patient interactions/clinical settings (e.g., replacing the word “patient” with “client,” who could be another genetic counselor or health professional).	3.89 (0.82)
	3.66 (0.89)
	0.23
	*p* = 0.010*
2. The ACGC PBCs should be expanded to include additional competencies used in the laboratory setting.	4.00 (0.75)
	3.90 (0.77)
	0.10
	*p* = 0.192
3. The ABGC board examination should include more questions focused on genetic counseling in the context of the laboratory setting.	3.76 (0.82)
	3.58 (0.90)
	0.18
	*p* = 0.038*
Section II—MGC program	
4. The knowledge and skills (competencies) that I learned in my Master's in Genetic Counseling Program are transferable to those that I use in my day‐to‐day work.	3.96 (0.80)
	4.21 (0.79)
	−0.25
	*p* = 0.001*
5. I use knowledge and skills (competencies) in my current work that I did not develop while enrolled in my master's in Genetic Counseling Program.	4.32 (0.70)
	4.32 (0.79)
	0.00
	*p* = 0.199
6. Additional didactic training (e.g., coursework) should be required within master's‐level genetic counseling programs to prepare graduates for positions in a laboratory setting.	3.63 (0.84)
	3.59 (0.89)
	0.04
	*p* = 0.583
7. Additional practice‐based training (e.g., rotations, field placements) should be required within master's‐level genetic counseling programs to prepare graduates for positions in a laboratory setting.	3.83 (0.87)
	3.69 (0.84)
	0.14
	*p* = 0.086
8. There should be the availability of separate tracks within master's‐level genetic counseling programs for those individuals who wish to pursue non‐clinical/non‐direct patient roles.	2.57 (1.17)
	2.45 (1.20)
	0.12
	*p* = 0.292
9. My Master's in Genetic Counseling Program was supportive of me considering work as a genetic counselor in a non‐direct patient care setting.	3.47 (1.04)
	3.90 (0.89)
	−0.43
	*p* < 0.001*
10. My master's in Genetic Counseling Program has provided me with sufficient knowledge and skills to perform variant interpretation.	2.50 (1.13)
	3.11 (1.21)
	−0.61
	*p* < 0.001*
11. My master's in Genetic Counseling Program has provided me with sufficient knowledge and skills to understand the limitations of various genomics‐based tests.	3.36 (1.01)
	3.80 (0.95)
	−0.44
	*p* < 0.001*
12. My master's in Genetic Counseling Program has provided me with sufficient knowledge and skills to understand the business of health care, such as insurance, billing, and reimbursement.	2.40 (1.05)
	2.66 (1.06)
	−0.26
	*p = 0*.013*
13. There have been times in my current position when I have had responsibilities which I believe were beyond my knowledge and skills as a master's level‐trained genetic counselor.	3.40 (1.14)
	3.30 (1.13)
	0.10
	*p* = 0.423
14. My master's in Genetic Counseling Program taught me how my competencies can be applied to roles outside of the clinical, direct patient care setting.	3.40 (1.05)
	3.85 (0.87)
	−0.45
	*p* < 0.001*
Section 3—Additional training	
15. Genetic counselors need additional training post‐master's degree to specialize as a laboratory genetic counselor.	2.53 (1.08)
	2.60 (1.07)
	−0.07
	*p* = 0.487
16. Genetic counselors need additional training post‐master's degree to be a clinical specialist genetic counselor (e.g., cancer, neurology, cardiology).	2.43 (1.03)
	2.35 (1.08)
	0.08
	*p* = 0.432
17. Post‐master's fellowship programs should be established to allow genetic counselors to train to become laboratory genetic counselors after graduation from their Master's in Genetic Counseling Program.	2.41 (1.06)
	2.64 (1.11)
	−0.23
	*p* = 0.033*
18. Academic, credit‐bearing certificate programs should be established to allow genetic counselors to train to become laboratory genetic counselors after graduation from their Master's in Genetic Counseling Program.	2.50 (1.13)
	2.71 (1.10)
	−0.21
	*p* = 0.057
19. Requiring additional training after graduate school in order to specialize would limit a genetic counselor's ability to change jobs.	4.45 (0.78)
	4.38 (0.82)
	0.07
	*p* = 0.341
Section 4—Identity	
20. Only genetic counselors who are interacting directly with patients to provide clinical care should be required to take the ABGC certification examination.	1.77 (1.02)
	1.67 (0.93)
	0.10
	*p* = 0.285
21. Only genetic counselors who are interacting directly with patients to provide clinical care should be required to be licensed.	2.56 (1.32)
	2.26 (1.24)
	0.30
	*p* = 0.021*
22. A person has to be providing genetic counseling directly with patients to be called a genetic counselor.	1.57 (1.01)
	1.56 (0.98)
	0.01
	*p* = 0.955
23. Even if I am no longer providing genetic counseling services directly with patients in a clinical setting, I still would identify as being a genetic counselor.	4.55 (0.79)
	4.48 (0.84)
	0.07
	*p* = 0.363
24. The proprietary nature of my work makes sharing my knowledge with others outside of my organization difficult.	2.56 (1.11)
	2.29 (1.02)
	0.27
	*p* = 0.013*
25. I am encouraged through my employer to volunteer my time to serve in professional organizations like the NSGC, ABGC, or ACGC.	3.78 (0.97)
	3.17 (1.13)
	0.61
	*p* < 0.001*
26. The NSGC supports my professional needs and other genetic counselors in my area of practice.	3.24 (1.01)
	3.54 (0.93)
	−0.30
	*p* = 0.002*
Section 5—Community of Practice	
27. I consider the Special Interest Groups (SIGs) of NSGC as a “community of practice.”	3.91 (0.80)
	4.00 (0.78)
	−0.09
	*p* = 0.272
28. I consider my place of employment as a “community of practice.”	4.04 (0.82)
	4.04 (0.80)
	0.00
	*p* = 0.933
29. Engaging in a community of practice is a beneficial method of training for genetic counseling specialization.	4.10 (0.73)
	4.23 (0.68)
	−0.13
	*p* = 0.085

* Significant (*p* < 0.05).

#### 3.2.1 Section 1. ACGC practice‐based competencies (PBCs) and ABGC certification examination

The majority of respondents (*n* = 268; 67.17%) agreed or strongly agreed that the ACGC PBCs that were approved in 2019 should be re‐written to better reflect competencies used outside of direct patient interactions/clinical settings, and the mean Likert score among genetic counselors working in the laboratory (M = 3.89, SD = 0.82) was significantly higher (*p =* 0.010) than the mean Likert score among genetic counselors working in other settings (M = 3.66, SD = 0.89). Similarly, most (*n* = 309; 77.44%) agreed or strongly agreed that the ACGC PBCs should be expanded to include additional competencies used in the laboratory setting, but the mean scores on the Likert scale for Lab and NonLab genetic counselors were not significantly different (*p* = 0.192). The majority (*n* = 247; 61.91%) agreed/strongly agreed that the ABGC examination should include more questions focused on genetic counseling in the context of the laboratory setting, with Lab genetic counselors (M = 3.76, SD = 0.82) having a significantly higher mean Likert score (*p* = 0.038) than NonLab genetic counselors (M = 3.58, SD = 0.90).

#### 3.2.2 Section 2. Master's in genetic counseling training programs

The vast majority (*n* = 327; 81.96%) of all respondents agreed or strongly agreed that the knowledge and skills learned in one's MGC program are transferable to those used in day‐to‐day work, but Lab genetic counselors (M = 3.96, SD = 0.80) had a significantly lower mean Likert score (*p* = 0.001) as compared to NonLab genetic counselors (M = 4.21, SD = 0.79). The majority agreed/strongly agreed that they use knowledge and skills in their current work which were not developed while enrolled in a MGC program (*n* = 355; 88.98%), that additional didactic training (e.g., coursework) should be required within MGC programs to prepare graduates for positions in a laboratory setting (*n* = 246; 61.17%), and that additional practice‐based training (e.g., fieldwork placements) should be required within MGC programs to prepare graduates for positions in a laboratory setting (*n* = 266; 66.67%); there were no significant differences in mean Likert scores between Lab and NonLab genetic counselors for these three items. A slight majority overall (*n* = 223; 55.89%) strongly disagreed/disagreed that there should be the availability of separate tracks within MGC programs for those individuals who wish to pursue non‐clinical/non‐direct patient roles, and there was no significant difference in mean Likert scores between Lab and NonLab genetic counselors.

Respondents were asked to note whether their MGC program had provided them with sufficient knowledge and skills suggested to be important in Schwartz et al. (2023). A large percentage (*n* = 177; 44.39%) of all respondents disagreed/strongly disagreed that their MGC program had provided them sufficient knowledge and skills in the area of variant interpretation, and genetic counselors working in the laboratory (M = 2.50, SD = 1.13) had a significantly lower mean Likert score (*p* < 0.001) than genetic counselors working in other settings (M = 3.11, SD = 1.21). Similarly, most disagreed/strongly disagreed (*n* = 216; 54.13%) they were sufficiently prepared to understand the business of health care, but again genetic counselors working in the laboratory (M = 2.40, SD = 1.05) had a significantly lower mean Likert score (*p* = 0.013) than genetic counselors working in other settings (M = 2.66, SD = 1.06). While most respondents agreed/strongly agreed (*n* = 244; 61.15%) that their understanding of limitations of various genomics‐based tests was sufficiently developed in their MGC program, genetic counselors working in the laboratory (M = 3.36, SD = 1.01) had a significantly lower mean Likert score (*p* < 0.001) than genetic counselors working in other settings (M = 3.80, SD = 0.95). A slight majority (*n* = 222; 55.64%) of all participants agreed/strongly agreed that there have been times in their current position when they have had responsibilities which they believed were beyond their knowledge and skills as a master's level‐trained genetic counselor, and there was no difference in mean Likert scores between Lab versus NonLab respondents.

Respondents were also asked to what extent they agreed that their MGC program was supportive of their considering work as a genetic counselor in a non‐direct patient care setting as well as whether or not their MGC program had taught them how their competencies can be applied to roles outside of the clinical, direct patient care setting. Most respondents agreed/strongly agreed that there was support for consideration of working in non‐patient care positions (*n* = 231; 57.93%), but genetic counselors working in the laboratory (M = 3.47, SD = 1.04) had a lower mean Likert score (*p* < 0.001) as compared to genetic counselors working in other settings (M = 3.9, SD = 0.89). Similarly, genetic counselors working in the laboratory (M = 3.40, SD = 1.05) had a significantly lower mean Likert score (*p* < 0.001) than genetic counselors working in other settings (M = 3.85, SD = 0.87) when asked if their MGC program taught them how their competencies could be applied to non‐direct patient care roles.

#### 3.2.3 Section 3. Additional training or credentialing for genetic counseling specialization

The majority (*n* = 207; 51.88%) strongly disagreed or disagreed that an additional post‐master's degree was needed to specialize as a genetic counselor in the laboratory or other clinical specialty (e.g., cancer, neurology, cardiology). There were no significant differences in the mean Likert scores between genetic counselors working within the laboratory or other settings for either of these survey items. A slight majority of all respondents (*n* = 203; 50.88%) strongly disagreed or disagreed that post‐master's fellowship programs should be established to allow MGC program graduates to work in the laboratory, with genetic counselors working in the laboratory (M = 2.41, SD = 1.06) having a significantly lower mean Likert score (*p* = 0.033) than genetic counselors working in other settings (M = 2.64, SD = 1.11). A slight minority (*n* = 191; 47.87%) of all respondents disagreed/strongly disagreed that post‐master's, academic, credit‐bearing certificates should be established to train genetic counselors to work in the laboratory setting, with 26.57% (*n* = 106) being neutral. There were no significant differences between mean Likert scores. The vast majority (*n* = 356; 89.22%) of all respondents strongly agreed/agreed that requiring additional training would limit the ability to change jobs, with no significant difference in mean Likert scores found between genetic counselors working in the laboratory and genetic counselors working in other settings.

#### 3.2.4 Section 4. Identity as a genetic counselor

The vast majority of all respondents strongly disagreed/disagreed that only genetic counselors working directly with patients should take the ABGC exam (*n* = 333; 83.46%) or that a genetic counselor has to be providing direct patient care to be called a genetic counselor (*n* = 343; 85.96%). There was no significant difference in mean Likert scores found between genetic counselors working within or outside of the laboratory for these questions. While the majority (*n* = 229; 57.39%) of all respondents strongly disagreed/disagreed that only genetic counselors who are interacting directly with patients to provide clinical care should be required to be licensed, genetic counselors working in the laboratory (M = 2.56, SD = 1.32) had a significantly higher mean Likert score (*p =* 0.021) than genetic counselors working in other areas (M = 2.26, SD = 1.24). Nearly all (*n* = 360; 90.22%) respondents would still consider themselves a genetic counselor even if they were no longer seeing patients, and there was no significant difference in mean Likert score between the groups. Interestingly, genetic counselors working in the laboratory (M = 3.78, SD = 0.97) had a significantly higher mean Likert score (*p* < =0.001) in response to the question as to whether they were encouraged by their employer to volunteer with professional organizations focused on genetic counseling, such as NSGC and ABGC, than were genetic counselors working in other settings (M = 3.17, SD = 1.13). However, genetic counselors working in the laboratory (M = 3.24, SD = 1.01) had a significantly lower mean Likert score (*p* = 0.002) when asked if they agree that NSGC supports their professional needs and others in their area of practice than genetic counselors working in other settings (M = 3.54, SD = 0.93).

#### 3.2.5 Section 5. Communities of practices (CoP) as a method for training for genetic counseling specialization

The vast majority of genetic counselors in all settings agreed or strongly agreed that they consider SIGs (*n* = 310; 77.69%) and their place of employment (*n* = 330; 82.71%) to be CoP and that engaging in a CoP is a beneficial method for genetic counselors to train to be specialized (*n* = 347; 86.97%). There were no differences in mean Likert scores found between genetic counselors working within or outside of the laboratory on these survey items.

## 4 DISCUSSION

This study was the second phase of an exploratory mixed methods study (QUAL→quant) to better understand how genetic counselors perceive how key components of genetic counselor training and credentialing reflect expanding roles for genetic counselors outside of the direct patient care setting such as the laboratory. However, unlike previous studies (Christian et al., 2012; Strohmeyer et al., 2023; Waltman et al., 2016), this study examined the perspectives of genetic counselors in a variety of practice settings while comparing these perspectives between those in the laboratory setting to those who were not.

The findings in our study align with previous literature focused on genetic counselors working in the laboratory (Goodenberger et al., 2015; Swanson et al., 2014; Waltman et al., 2016). In our study, genetic counselors working in the laboratory reported being significantly less supported and prepared by their MGC program to perform roles commonly used in the laboratory setting, including variant interpretation, limitations of genomic‐based tests, and the business of health care, than those working outside of the laboratory setting. However, it is possible that genetic counselors not working in the laboratory setting were less likely to be regularly performing these roles, and thus support and preparation for these roles were not of as much a concern as they were for genetic counselors in laboratory roles.

Swanson et al. (2014) proposed that master's‐level genetic counseling programs update their curricula to prepare graduates for positions in the laboratory setting. Our study found that genetic counselors across practice areas, both in and outside of laboratory settings, agreed or strongly agreed that additional didactic and practice‐based training focused on the competencies and roles of genetic counselors in the laboratory should be required elements of genetic counseling programs' curricula. However, how MGC programs currently incorporate these additional needed competencies is unclear (Riconda et al., 2018).

During the study period, a draft of revised ACGC PBCs were shared publicly for feedback, and revised ACGC PBCs were released in September 2023. Notably, the 2023 PBCs were revised to apply to a variety of practice settings, including the laboratory and industry. For example, the 2019 ACGC PBCs frequently used the term patient, but the 2023 PBCs use the term client instead, which is defined as “individuals or groups who utilize or receive genetic counseling services” (ACGC, 2023), which can be interpreted to include the population with whom genetic counselors work outside of the direct patient‐care setting such as other health professionals and business partners. By June 2025, MGC programs will need to review their curricula to ensure they are in compliance with these new ACGC PBCs, including those focused on genetic testing methodologies, variant interpretation, and financial considerations in the delivery of genetic services (ACGC, 2023). Re‐assessment of genetic counselors' perceptions of their preparation for these competencies will be warranted in the future.

The vast majority of all respondents agreed or strongly agreed that the previous ACGC PBCs (2019) should be expanded to include additional competencies used in the laboratory setting, a sentiment that supports the recommendation made by Waltman et al. (2016). The updated 2023 ACGC PBCs address a number of competencies related to, but not exclusively used in, the laboratory setting, including the aforementioned variant interpretation, genetic testing methodologies, and financial considerations in genetic service delivery (ACGC, 2023). Goodenberger et al. (2015) emphasized the transferability of advanced counseling skills to the various roles of genetic counselors working in the laboratory and urged master's in genetic counseling programs to consider expanding practical training of genetic counselors to the laboratory setting, which in turn would enhance their understanding of how their training can be applied in this setting.

Baty et al. (2016) described the work of the Committee on Advanced Training for Certified Genetic Counselors (CATCGC), which was charged by the Genetic Counselor Educators Association [GCEA; formally known as the Association of Genetic Counseling Program Directors (AGCPD)] to explore and propose a model by which genetic counselors can advance their careers through post‐master's training and career development. They acknowledged that:

As genomic discoveries continue to expand into clinical care there will be new opportunities for genetic counselors to integrate into routine health care. New opportunities may require new or expanded competencies. As such, there is a need to strategically develop educational and training opportunities geared towards current and future roles and responsibilities (p. 630).

However, in our study, participants working both within and outside the laboratory setting disagreed that post‐master's degrees or certifications should be required to work in the laboratory or other specialty areas as they felt requirements could restrict movement into these positions. Therefore, as supported by other findings of our study and by others (Amendola et al., 2021; Riconda et al., 2018; Swanson et al., 2014), the addition of both didactic and practice‐based training focused on the non‐direct patient roles of genetic counselors in the laboratory setting should be incorporated into master's‐level genetic counseling programs.

We recognize that inclusion of additional content into MGC programs is a daunting task, given the considerable amount of material needed to be taught within what is most often a 21–24‐month period. As critical drivers of genetic counselor education, directors of MGC programs must be engaged in discussions regarding how entry‐level genetic counselors may be well‐prepared to enter an increasingly diverse workplace. Baty and Davis (2023) conducted semi‐structured interviews with directors of MGC programs in North America who noted the benefits and complexity of aligning their programs' goals and development with ACGC PBCs and Standards. Most program directors noted that regular revision of the ACGC PBCs ensure that training programs are keeping up to date with new developments in the field. Yet a significant number of program directors expressed that the need to conform to a national set of standards limited the flexibility needed to account for the realities of unique and diverse programs.

However, the competencies used within the laboratory setting, as found by Schwartz et al. (2023) and many others, including Goodenberger et al. (2017) and Hart et al. (2023), are applicable across multiple settings in which genetic counselors work, not specifically the laboratory. Therefore, instruction in support of development of these competencies may not require addition of new, separate courses but rather could be integrated into existing coursework. Murphy et al. (2023) found that MGC program directors were amenable to sharing curricula, and thus new content and delivery methods to incorporate laboratory‐related competencies could be developed by select MGC programs and shared broadly.

To address integration of the new ACGC PBCs into fieldwork experiences and increase exposure to genetic counselor roles outside of clinical settings, consideration should be made as to whether supervised cases to be included in the documentation for ABGC certification eligibility necessitate direct patient care cases. In the meantime, the current culture that students should acquire significantly more than the requisite 50 core cases should be changed. Instead MGC program directors can encourage or require that students participate in supplemental rotations that expose them to the expanding non‐direct patient care roles and practice settings of genetic counselors, including the laboratory/industry, research, public health, and education. This shift could be accomplished by shortening required participatory rotations to ensure students focus on the diversity of core cases rather than the number, thus allowing for more time in the clinical phase of training for supplemental rotations. This recommendation is supported by past research that has found a plateau of genetic counseling students' reported self‐efficacy after 80–100 core cases have been documented (Owens‐Thomas et al., 2019). Additionally, increasing the availability and making a requirement of laboratory field placements may address the shortage of training sites, particularly in geographic areas where there are several MGC programs.

In addition to examining perceptions of preparation and additional training for roles in the laboratory, the survey addressed unexpected findings from Schwartz et al. (2023) that focused on the concepts of professional identity and communities of practice. Genetic counselors in industry and laboratory settings report struggling with their professional identity as genetic counselors, often due to perceived negative feelings and stigmatization from their genetic counselor colleagues in other settings (Groepper et al., 2015; Strohmeyer et al., 2023; Zetzsche et al., 2014). Interestingly, our study found that genetic counselors working in both laboratory and non‐laboratory settings agreed that genetic counselors in non‐direct patient care positions should identify as a genetic counselor. This is contradictory to how genetic counselors working in the laboratory have reported their perceptions by their colleagues in non‐laboratory settings and in turn their expressed struggles with the genetic counselor identity (Strohmeyer et al., 2023). Additional studies are necessary to decipher where this disconnect occurs between expression of genetic counselor identity support versus perceived identity support.

Further integration of genetic counselors working in the laboratory, by inviting them to teach in didactic coursework and adding more required supplemental rotations into field placements, may support the professional identity of genetic counselors working in these settings, particularly those working remotely, and engender a greater sense of belonging in the profession (van Lankveld et al., 2021). Gerrity et al. (1997) summarized literature showing that physicians who serve as clinician‐educators have increased job and career satisfaction and reap a number of rewards from teaching the next generation of health professionals, including intellectual and professional growth, and a renewed sense of importance in their work. Reiser (2019) noted that both the NSGC Code of Ethics and the PBCs compel practicing genetic counselors to support the expansion and training of the next generation of genetic counselors, and in turn, contribute to the professional development of genetic counselors who serve in these roles. How teaching and supervision within MGC programs may be supported and impact the professional identity of genetic counselors working in non‐direct patient care roles is worthy of further exploration (van Lankveld et al., 2021).

### 4.1 Practice implications

During MGC programs, more exposure to the diverse roles of genetic counselors in the laboratory setting, including variant interpretation, understanding of limitations of genomic tests, and the business of health care, is desired by genetic counselors working both within and outside of the laboratory setting, echoing recommendations from others (Amendola et al., 2021; Arjunan et al., 2021; Goodenberger et al., 2015; Swanson et al., 2014; Waltman et al., 2016). The inclusion of cases involving non‐direct patient care for documentation of eligibility for ABGC certification, which would require revisiting the ACGC Standards for MGC training programs (ACGC, 2019b), should be further debated within the greater genetic counselor community. Of concern, only a slight majority of genetic counselors surveyed agreed or strongly agreed that NSGC supports their professional needs.

### 4.2 Limitations and future research

A primary limitation of the study was the low response rate (7.31%) and thus small sample size (*N* = 399). Comparison of demographics among survey respondents to the 2021 NSGC PSS showed that they are representative of the broader population of genetic counselors, although genetic counselors working in laboratory settings and non‐direct patient care roles were over‐represented in the survey sample. The survey was created by the research team, and while pilot tested and subject to expert review, was not formally validated.

The survey was administered prior to the revised ACGC PBCs being made effective in September 2023 (ACGC, 2023), and therefore, responses to some survey questions regarding desire to change the wording of the PBCs may be answered differently if the survey had been administered after September 2023 since, as noted above, the 2023 ACGC PBCs use the term client rather than patient and specific include knowledge of genetic testing methodologies, variant interpretation, and the financial considerations of genetic service delivery (ACGC, 2023). An exploration of genetic counseling students' perceptions regarding the value and impact of integrating more laboratory field placements while reducing direct patient care training is another potential area of future research. The survey findings about professional identity and CoP were consistent with those found in the qualitative phase of the study by Schwartz et al. (2023). Future research focused on these important concepts is currently underway with funding from the NSGC Jane Engelberg Memorial Fellowship (JEMF).

## 5 CONCLUSIONS

Overall genetic counselors across diverse practice settings agree that the training, competency evaluation, and certification of genetic counselors need to be revised and expanded to include competencies used by genetic counselors in non‐direct patient care roles, including in the laboratory. Post‐master's academic training such as a certificate was not a preferable method to gain additional competencies, as they may limit movement into new roles for genetic counselors or shifts between existing practice settings (e.g., prenatal to oncology). As identified in the first phase of the study by Schwartz et al. (2023), and aligned with previous literature, emphasis on the knowledge, skills, and application of ACGC PBCs used by genetic counselors in the laboratory setting should be incorporated into didactic and field training of MGC programs. Our findings suggest that the organizations focused on the field of genetic counseling (e.g., NSGC, ACGC, and ABGC) must continue to gauge the perspectives and needs of their stakeholders to ensure they are aligned with the rapidly changing landscape of the genetic counseling profession.

## AUTHOR CONTRIBUTIONS

The primary author (LS) designed the research study, created and administered the survey in Qualtrics©, and performed all statistical analyses. She is a genetic counselor by training, however, is no longer in clinical practice since completing her doctorate in higher education administration and joining The George Washington University as a full‐time faculty member. Other authors (MM, AA, MG, RM, and SW) are currently practicing genetic counselors working in either commercial (MM, AA, and SW) or academic (MG) laboratories or are affiliated with a master's in genetic counseling training program (RM). They are active members of the National Society of Genetic Counselors (NSGC) and served on an advisory board for the ABGC‐funded study. They assisted in development of the survey instrument, debriefing of findings identified through data analysis, and preparation of this manuscript. CH is a second‐year medical student who contributed to the data analysis and creation of the figure in the preparation of the manuscript.

Author LS confirms that she had full access to all the data in the study and takes responsibility for the integrity of the data and the accuracy of the data analysis. All of the authors gave final approval of this version to be published and agreed to be accountable for all aspects of the work in ensuring that questions related to the accuracy or integrity of any part of the work are appropriately investigated and resolved.

## CONFLICT OF INTEREST STATEMENT

LS, MG, CH, and RM declare that they have no conflict of interest. MM is a full‐time employee and stockholder at Natera, Inc. AA is a full‐time employee of GRAIL LLC, a subsidiary of Illumina and owns stock in Illumina, Myriad Genetics, Invitae, and GeneDx. SW is a full‐time employee of BioReference Health, LLC and owns stock in BioReference Health LLC and Quest Diagnostics.

## ETHICS STATEMENT

Human studies and Informed Consent: This study was reviewed and granted an exemption by The George Washington University Institutional Review Board (NCR203188). All procedures followed were in accordance with the ethical standards of the responsible committee on human experimentation (institutional and national) and with the Helsinki Declaration of 1975, as revised in 2000. Implied informed consent was obtained for individuals who voluntarily completed the online survey and submitted their responses.

Animal Studies: No non‐human animal studies were carried out by the authors for this article.

## Supporting information


Data S1:

## Data Availability

The data that support the findings of this study are available on request from the corresponding author. The data are not publicly available due to privacy or ethical restrictions.
