# Supplementary material for: Graduate training, credentialing, and continuing education to prepare genetic counselors for laboratory roles—Results of a national survey
Source: J Genet Couns. 2024 Feb 9;34(1):e1883. doi: 10.1002/jgc4.1883 (PMC11726605; doi:10.1002/jgc4.1883)
Supplement: Supplementary file 1 — Data S1: [file JGC4-34-0-s001.docx]

# Informed Consent

**Informed Consent Form**

**Phase II - Exploration of Need for Additional Credentialing for Laboratory-based Genetic Counselors Principal Investigator: Lisa Schwartz, EdD., 571-553-0137**

**INTRODUCTION**

**You are invited to take part in a research study being conducted by Lisa Schwartz, EdD, MS of the George Washington University School of Medicine and Health Sciences Department of Biomedical Laboratory Sciences.**

**You are being asked if you want to take part in Phase II of this study because you are a diplomat of the American Board of Genetic Counseling. Please read this form and ask me any questions that will help you decide if you want to be in the study. Taking part is completely voluntary and even if you decide you want to, you can quit at any time.**

**PURPOSE**

**The expanded options of genetic and genomic testing have resulted in more genetic counselors working in the clinical laboratory. However, the practice-based competencies – the knowledge and skills - needed among entry-level genetic counselors to address the many additional issues found in the laboratory has not been thoroughly explored. Phase I of this two-phase study investigated what genetic and non- genetic professionals working in a laboratory setting believe are the specific knowledge, skills, and competencies that are not currently listed in the Accreditation Council for Genetic Counseling Practice-based**

**Competencies as they relate to the practice of laboratory genetic counselors. After analyzing the data collected through Phase I, this survey was developed to assess current genetic counselors’ perceptions of their competency in these areas. The study's findings will help the American Board of Genetic Counseling understand the methods to assist genetic counselors in gaining this knowledge and skills so that they are better prepared to enter the laboratory setting and support public health.**

**PROCEDURES**

**If you agree to participate, you will be asked to complete an online survey, which will take approximately 15 minutes to complete.**

**RISKS & CONFIDENTIALITY**

**The risk to study participants is minimal. It is possible, although unlikely, that your survey responses may be linked to you.**

**The following steps are being taken to reduce these risks:**

**Your name and any other identifying information will not be collected by the electronic survey.**

**Upon completion of the survey, you will be directed to a new form, which is not linked to your survey responses, to provide your email address to be entered into a raffle for one of twenty (20)-$50 gift cards.**

**At the conclusion of the study, all data collected in the online survey and raffle collection form will be destroyed.**

**Your records for the study may be reviewed by departments of the University responsible for overseeing research safety and compliance.**

**BENEFITS**

**Taking part in this research will not help you directly, however the benefit to society will be informing leaders in the organization that provides credentialing to genetic counselors, the American Board of Genetic Counselors, as well as genetic counseling training programs, of the knowledge, skills and practice-based competencies needed among entry- level genetic counselors, as perceived by professionals already working in the laboratory setting, and the degree to which genetic counselors working in different settings believe there may be gaps in these knowledge, skills and competencies and preferred ways in which these can be obtained through continuing education of genetic counselors.**

**COMPENSATION**

**As a token of appreciation for your participation in the study, upon completion of the survey, you will be directed to a form, which is not linked to your survey responses, to provide your email address to be entered**

**into a raffle for one of twenty (20)-$50 gift cards.**

**QUESTIONS**

**Talk to the research team if you have questions, concerns, complaints, or think you have been harmed. You can contact the Principal Investigator listed on the front of this form at (571) 553-0137. For questions regarding your rights as a participant in human research call the GWU Office of Human Research at 202-994- 2715.**

**Your willingness to participate in this research study is implied if you proceed.**

**Please feel free to print a copy of this consent form in case you want to read it again or call someone about the study.**

**Introduction:**

**Exploration of Need for Additional Credentialing for Laboratory- based Genetic Counselors**

## **Introduction:** The purpose of this survey is to assess the perceptions among genetic counselors working in a variety of practice areas of their preparation, and perceived need for additional training, to work in the laboratory setting. The results of this survey will help inform the development of educational activities to support genetic counselors working in these roles.

**Definitions:**

**Laboratory genetic counselor:** an individual who has completed a master’s degree in genetic counseling and who is employed by a private company or medical facility that offers laboratory genetic services, such as testing or treatment. A laboratory genetic counselor is primarily serving as an intermediary between the laboratory and healthcare providers who are utilizing genetic services, and typically not working directly with patients in a clinical setting, but may serve in a variety of roles, including but not limited to test utilization management and support, sales and marketing, product development, and variant interpretation.

**ACGC Practice-based Competencies (PBCs):** the twenty-two (22) competencies (applicable knowledge and skills) published by the Accreditation Council for Genetic Counseling (ACGC) that an entry-level provider must demonstrate to successfully practice as a genetic counselor. The PBCs inform both the American Board of Genetic Counseling (ABGC) certification examination, as well as the required didactic and experiential components of ACGC-accredited genetic counseling training programs. (<https://www.gceducation.org/practice-based-competencies/>)

**Block 1**

**Section 1 - The following questions focus on the ACGC Practice-based Competencies (PBCs) and ABGC certification examination.**

## To what extent do you disagree or agree with the following statements:


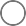

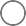

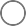

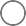

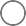

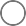

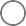

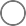

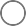

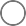

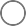

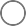

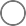

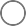

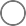


Strongly

Disagree Disagree Neutral

Agree

Strongly Agree

1. The ACGC PBCs should be re-written to better reflect competencies used outside of direct-patient interactions/clinical settings (e.g., replacing the word ‘patient’ with ‘client’, who could be another genetic counselor or health professional).
2. The ACGC PBCs should be expanded to include additional competencies used in the laboratory setting.
3. The ABGC board examination should include more questions focused on genetic counseling in the context of the laboratory setting.

**Block 2**

**Section 2 - The following questions focus on the your perceptions of Master’s in Genetic Counseling Training Programs**

To what extent do you disagree or agree with the following statements:


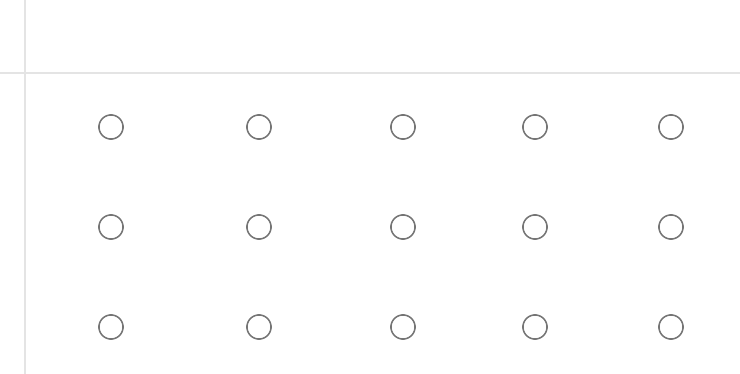


Strongly

Disagree Disagree

Neutral

Agree

Strongly Agree

1. The knowledge and skills (competencies) that I learned in my Master's in Genetic Counseling Program are transferable to those that I use in my day-to-day work.
2. I use knowledge and skills (competencies) in my current work that I did **not** develop while enrolled in my Master's in Genetic Counseling Program.
3. Additional **didactic** training (e.g., coursework) should be required within Master’s-level genetic counseling programs to prepare graduates for positions in a laboratory setting.
4. Additional **practice-based training** (e.g., rotations, field placements) should be required within Master’s-level genetic counseling programs to prepare graduates for positions in a laboratory setting.
5. There should be the availability of **separate tracks** within master’s-level genetic counseling programs for those individuals who wish to pursue non-clinical/non-direct patient roles.
6. My Master's in Genetic Counseling Program was supportive of me considering work as a genetic counselor in a non-direct patient care setting.
7. My Master's in Genetic Counseling Program has provided me with sufficient knowledge and skills to perform variant interpretation.
8. My Master's in Genetic Counseling Program has provided me with sufficient knowledge and skills to understand the limitations of various genomics-based tests.
9. My Master's in Genetic Counseling Program has provided me with sufficient knowledge and skills to understand the business of healthcare, such as insurance, billing, and reimbursement.
10. There have been times in my current position when I have had responsibilities which I believe were beyond my knowledge and skills as a master’s level-trained genetic counselor.
11. My Master’s in Genetic Counseling Program taught me how my competencies can be applied to roles **outside** of the clinical, direct-patient care setting.

Strongly

Disagree Disagree Neutral Agree


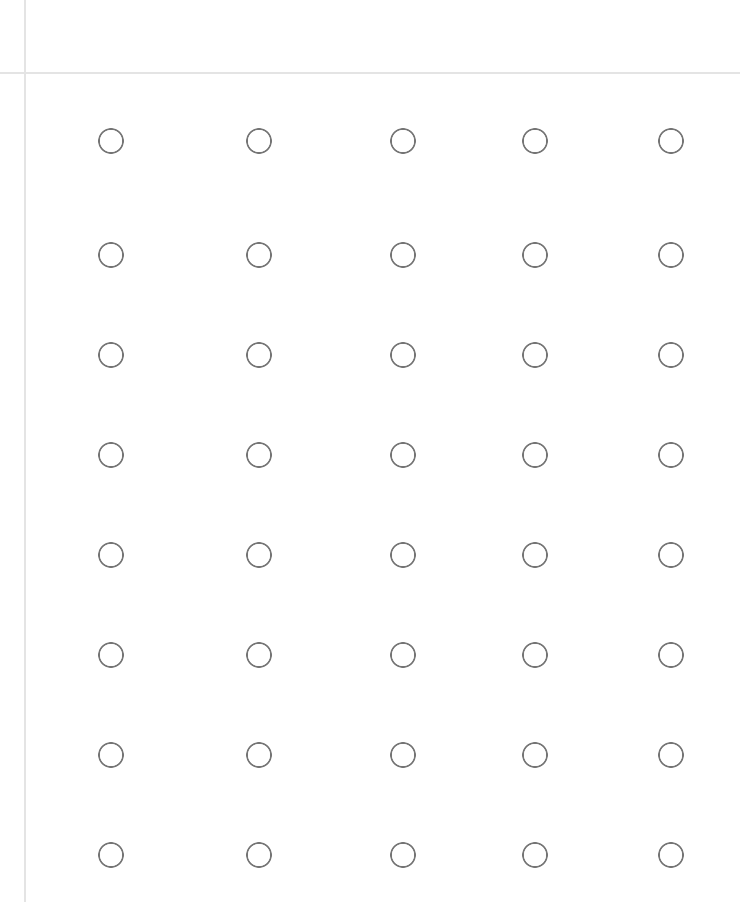


Strongly Agree

# Block 3

**Section 3 - The following questions focus on additional training or credentialing for genetic counseling specialization.**

### To what extent do you disagree or agree with the following statements:

Strongly Disagree Disagree Neutral Agree Strongly Agree


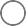

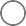

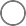

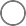

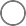


1. Genetic counselors need additional training post- master’s degree to specialize as a **laboratory** genetic counselor.


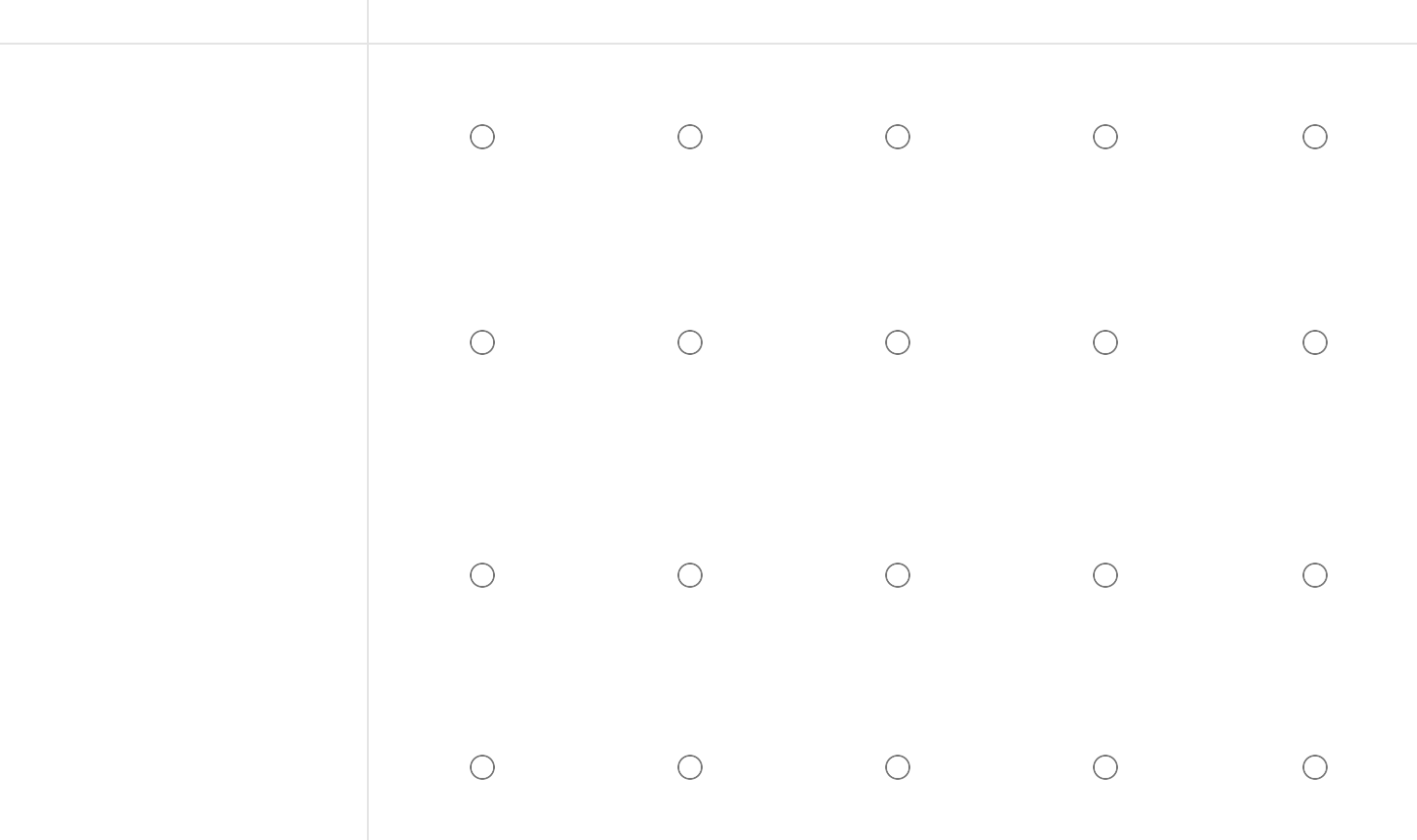
Strongly Disagree Disagree Neutral Agree Strongly Agree

1. Genetic counselors need additional training post- master’s degree to be a **clinical specialist** genetic counselor (e.g., cancer, neurology, cardiology).
2. Post-master’s fellowship programs should be established to allow genetic counselors to train to become laboratory genetic counselors **after graduation** from their Master's in Genetic Counseling Program.
3. Academic, credit-bearing certificate programs should be established to allow genetic counselors to train to become laboratory genetic counselors **after graduation** from their Master's in Genetic Counseling Program.
4. **Requiring** additional training after graduate school in order to specialize would limit a genetic counselor’s ability to change jobs.

# Block 4

**Section 4 - The following questions focus on one’s perception of identity as a genetic counselor**

### To what extent do you disagree or agree with the following statements:

Strongly Disagree Disagree Neutral Agree Strongly Agree


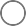

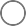

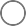

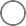

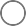


1. Only genetic counselors who are interacting directly with patients to provide clinical care should be required to take the ABGC certification examination.


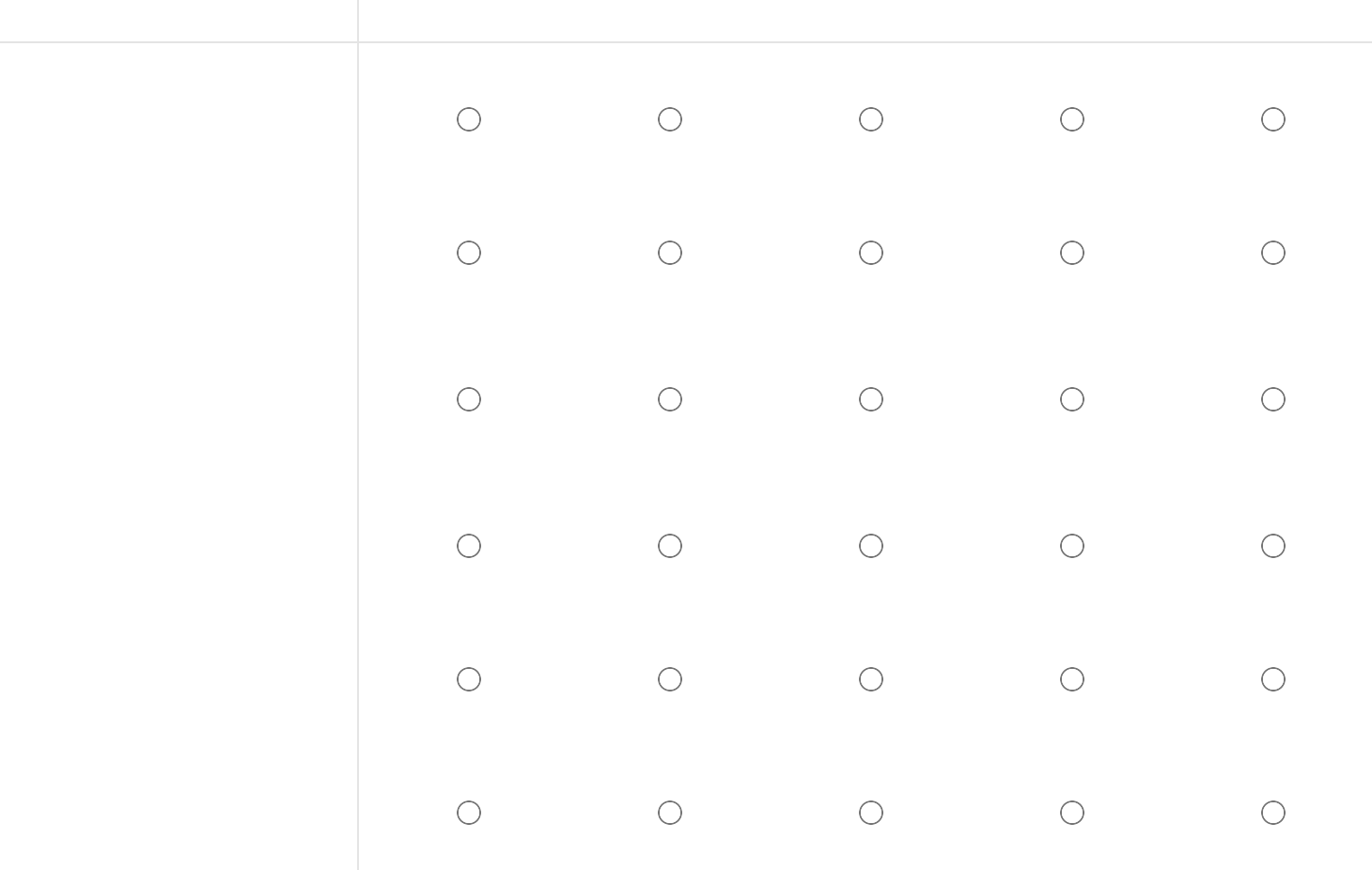
Strongly Disagree Disagree Neutral Agree Strongly Agree

1. Only genetic counselors who are interacting directly with patients to provide clinical care should be required to be licensed.
2. A person has to be providing genetic counseling directly with patients to be called a genetic counselor.
3. Even if I am no longer providing genetic counseling services directly with patients in a clinical setting, I still would identify as being a genetic counselor.
4. The proprietary nature of my work makes sharing my knowledge with others outside of my organization difficult.
5. I am encouraged through my employer to volunteer my time to serve in professional organizations like the NSGC, ABGC, or ACGC.
6. The NSGC supports my professional needs and other genetic counselors in my area of practice.

# Block 5

**Section 5 - The following questions focus on the concept of communities of practices as a method for training for genetic counseling specialization.**

**Communities of Practice - A “community of practice” is a group of people who share a concern or passion for something they do, share information and build relationships, and develop shared resources for practice.**

### Considering that definition to what extent do you disagree or agree with the following statements:

Strongly Disagree

Disagree

Neutral

Agree

Strongly Agree

Strongly Disagree Disagree Neutral Agree Strongly Agree


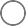

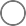

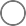

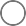

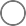

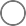

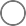

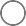

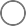

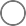

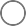

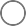

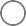

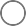

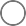


1. I consider the Special Interest Groups (SIGs) of NSGC as a “community of practice”.
2. I consider my place of employment as a “community of practice”.
3. Engaging in a community of practice is a beneficial method of training for genetic counseling specialization.

# Section 6 - Demographics

## Indicate the academic degrees that you have completed or are in the process of completing:

Yes No


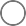

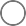

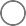

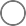

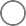

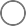

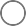

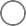

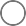

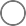

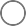

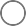

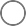

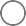

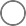

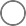

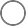

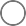


Master’s degree (or equivalent) in Genetic Counseling

Master’s degree (or equivalent) in another life science-based field (e.g., medical laboratory sciences, microbiology)

Master’s degree in Public Health (MPH) Master’s degree in Education

Master’s degree in Business (MBA) or equivalent Medical degree (MD or DO)

Doctoral degree in life-science-based field (e.g., biochemistry, genetics) Doctoral degree in education

Other (please specify)

## I have participated in the following activities to further develop the competencies needed to perform my job since completing my Master’s in Genetic Counseling Program:

Yes

No

Yes No


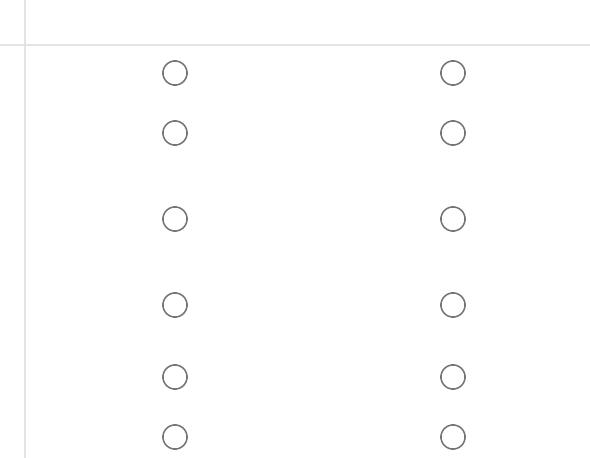


Academic, credit-bearing degree (e.g., PhD, MBA, MPH)

Academic, credit-bearing coursework (e.g., statistics, research methods, education)

Continuing Education – as offered by NSGC, SIG or other professional organization or academic organization that is not academic/credit-bearing or offered by place of employment

Work-based – structured training offered by place of employment (e.g., orientation, onboarding)

Informal on the job training – apprenticeship model; learning from others in same or different roles within workplace

On my own (e.g., literature review, Internet searches)

## Within which of the following years did you graduate from a genetic counseling program?


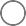
 2020-2021


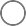
 2010-2019


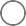
 2000-2009


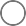
 1990-1999


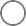
 1980-1989


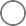
 1970-1979


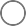
Not applicable; I did not attend a genetic counseling program

## Please indicate which of the following applies to you:


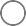
 ABGC-certified AND licensed


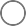
 ABGC-certified but NOT licensed
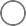
 ABGC eligible


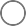
Not applicable

## Choose one that best describes your **CURRENT** position:


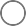
 Direct patient care


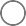

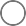
 Non-direct patient care Mixed position

## Please indicate your **CURRENT** primary employer work setting:

NOTE: This list was amended from the 2021 NSGC Professional Status Survey and is in alphabetical order.


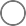
 Diagnostic Laboratory –Non-commercial, Academic
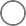
 Diagnostic Laboratory – Commercial, Academic


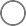
 Diagnostic Laboratory – Commercial, Non-academic
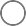
 Government Organization or Agency


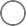
 Hospital/Medical Facility – Academic Medical Center


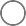
 Hospital/Medical Facility – Private (nonprofit or for profit)
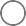
 Hospital/Medical Facility - Public


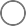
 Insurance Company/Benefit Management Company
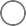
 Pharmaceutical Company


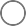
 Physicians Private Practice


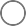
 Private Company (specify type)


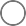
 Self-employed/Private Practice
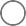
 University


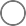

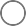
 Other work setting (specify type) Not currently employed

1. What is your **CURRENT** job title?

## NOTE: This list was amended from the 2021 NSGC Professional Status Survey and is in order of most commonly reported.


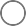
 Genetic Counselor


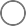
 Genetic Counselor, Senior/Lead/Supervisor/Coordinator
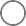
 Medical Science Liaison


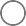
 Laboratory Counselor/ Coordinator/Support


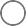
 Manager, Clinical Services/Genetic Services/Genetic Counseling
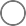
 Director- other


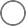
 Professor/Instructor/Assistant Professor/Associate Professor


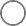
 Director, Clinical Services/ Genetic Services/Genetic Counseling Research Counselor/Scientist/Assistant/Associate

Director, Genetic Counseling Training Program Manager- other

Assistant Director/Coordinator, Genetic Counseling Training Program Assistant Director- other

Project Manager/Project Director

Manager, Laboratory Genetic Services Product Manager/Product Specialist Other job title (specify)

Not currently employed

1. Identify all of your roles within your **CURRENT** position (**mark all that apply**):

## NOTE: This list was amended from the 2021 NSGC Professional Status Survey and is in order of most commonly reported.

Patient-facing/Direct Patient Care Education/Teaching

Supervision - Students Coordination - Clinical Research

Supervision - Employees

Insurance Prior Authorization/ Appeals Program Development

Writing - Education/Patient Material Management - Administrative

Writing - Scientific

Coordination - Research

Laboratory - Support/Customer Service Clerical Laboratory - Report Writing

Customer Liaison

Business Development

Laboratory - Variant Interpretation Recruiting/Hiring

Advocacy

Management - Project Utilization Management

Product Development/Management IRB/Regulatory

Financial/Contract/Budget Development or Management Laboratory - Field Support

Marketing (of services, products, etc. ) Writing - Grant

Management - Grant Laboratory - Sales

Writing - Media/Press Public Policy

Other roles (please specify) Not applicable - unemployed

## Designate your **CURRENT** primary area of practice:

NOTE: This list was amended from the 2021 NSGC Professional Status Survey and is in order of most commonly reported.

Cancer Genetics - Adult Prenatal

Pediatrics

Molecular/Cytogenetics/Biochemical Testing Genomic Medicine

Cardiology

Neurogenetics

Preconception/Reproductive Screening Metabolic Disease

Preimplantation Genetic Testing, ART/IVF, Infertility General Adult Genetics

Other practice area (please specify) Not applicable - unemployed

1. Indicate total # of years employed in your **CURRENT position**

Less than 1 year

1-4 years

5-9 years

10-14 years

15-19 years

20-24 years

More than 25 years

1. Indicate total # of years employed in your **CURRENT area of practice**

Less than 1 year 1-4 years

5-9 years

10-14 years

15-19 years

20-24 years

More than 25 years

## For the following statements, please choose **one** which best describes your **current** position:

Yes No Not sure

My current position required that I graduated from an accredited Master’s in Genetic Counseling Program.

My current position required that I be board certified as a genetic counselor.

My current position required that I be licensed as a genetic counselor.

Yes No Not sure

My current position required that I had clinical experience after completing my Master's in Genetic Counseling Program
